# State of Health Estimation Based on the Long Short-Term Memory Network Using Incremental Capacity and Transfer Learning

**DOI:** 10.3390/s22207835

**Published:** 2022-10-15

**Authors:** Lei Yao, Jishu Wen, Shiming Xu, Jie Zheng, Junjian Hou, Zhanpeng Fang, Yanqiu Xiao

**Affiliations:** 1 Henan Engineering Research Center of New Energy Vehicle Lightweight Design and Manufacturing, Zhengzhou University of Light Industry, Zhengzhou 450001, China; 2 Henan Key Laboratory of Intelligent Manufacturing of Mechanical Equipment, Zhengzhou University of Light Industry, Zhengzhou 450001, China

**Keywords:** lithium-ion battery, SOH estimation, discrete wavelet transform, grey relation analysis, long-short term memory neural network, transfer learning

## Abstract

Battery state of health (SOH) estimating is essential for the safety and preservation of electric vehicles. The degradation mechanism of batteries under different aging conditions has attracted considerable attention in SOH prediction. In this article, the discharge voltage curve early in the cycle is considered to be strongly characteristic during cell aging. Therefore, the battery aging state can be quantitatively characterized by an incremental capacity analysis (ICA) of the voltage distribution. Due to the interference of vibration noise of the test platform, the discrete wavelet transform (DWT) methods are accustomed to soften the premier incremental capacity curves in different hierarchical decompositions. By analyzing the battery aging mechanism, the peak of the curve and its corresponding voltage are used in the characterization of capacity decay by grey relation analysis (GRA) and to optimize the input of the deep learning model, and finally, the double-layer long short-term memory network (LSTM) model is used to train the data. The results demonstrate that the proposed model can predict the SOH of a single battery cycle using only small batch data and the relative error is less than 2%. Further, by freezing the LSTM layer for transfer learning, it can be used for battery health estimation in different loading modes. The results of training and verification show that this method has high accuracy and reliability in SOH estimation.

## 1. Introduction

The safety of the lithium-ion battery itself is critical in its practical application. Suppose battery failure occurs in the process of use. In that case, it may lead to performance degradation or loss of the corresponding power equipment or system, which will increase the risk of thermal runaway and even cause personal injury or death [1]. Therefore, as an ideal energy storage device, it is of extensive practical significance to find a method to accurately monitor the SOH of lithium-ion batteries [2]. There are many kinds of research on the life prediction of lithium ions in related fields [3,4,5]. In terms of research methods, the remaining life prediction methods of lithium-ion batteries can be approximately divided into two categories: model-driven and data-driven methods [6].

The model-based methodology is relatively mature and widely used in engineering practice. The method mainly models the battery according to the elder law and simulates the battery behavior [7,8,9]. There are several different forms, for instance, the electrochemical impedance spectroscopy model. Wang et al. [10] proposed a model-based estimation method to evaluate the insulation state of the battery pack. Through the principle of electro-engineering, the running state of the battery is simulated to estimate the future running condition of the storm. Galeotti et al. [11] analyzed the electrochemical impedance spectroscopy (EIS) of the battery and found that in continuous use, the ohmic resistance and available capacity increased linearly with the rapid aging of the battery. The stable relationship can be directly used to measure the battery’s performance and determine the battery’s service life and health status. It is believed that EIS measurement can indirectly obtain charge transfer resistance and directly obtain various measurement parameters such as ohmic resistance, solid electrolyte interface (SEI) resistance, and double-layer capacitance. The parameters can be used to improve the equivalent circuit model of batteries. The accuracy of life prediction is further enhanced. Plett [12] proposed the extended Kalman filter for battery state estimation and proved its accuracy and engineering practicability for quantitative analysis. 

Similarly, Yang et al. [13] proposed a battery SOH estimation method based on the adaptive double-extended Kalman filter, which used the double-extended Kalman filter for online estimation and combined the adaptive algorithm and fuzzy controller correct the noise. The dynamic stress test condition experiment verifies it. This method can estimate the battery state. The method-based model is based on the lithium-ion battery degradation and failure mechanism to accomplish the SOH estimation and prediction, although able to represent the aging condition of internal model attenuation rules of critical parameters to achieve the intention of the SOH estimation. Still, with the development of technology, generalization is relatively weak, and prediction and analysis precision also need to be improved.

Compared with the model-based method, the accuracy relies on the accuracy of parameter identification and the complexity of the model, so it is not easy to achieve high reliability and accuracy. With the development of data mining and computing power, the data-driven method has become a hot discussion for more researchers [14,15,16]—examples include the support vector machine [17], the neural network [18], the extreme learning machine [19], random forest [20], and Gaussian filtering [21].

The data-driven method can process many complex nonlinear system data and has many advantages, such as no need for detailed information on battery characteristics. In addition, some algorithms have a strong generalization and can accurately predict SOH under different working conditions [22]. Wang et al. [23] used a support vector machine to deduce correlation vectors that could be used for training, and combined with the conditional three-parameter model to fit the predicted values of correlation vectors to indicate battery life. Li et al. [24] proposed an online health assessment method for lithium batteries based on the support vector machine (SVM) and particle swarm optimization (PSO), using PSO to optimize the SVM kernel function and combining state of charge (SOC) to estimate SOH. It provides a reference for online battery detection and SOH estimation. Compared with support vector machines, the neural network has a more vital self-learning ability and can obtain higher prediction accuracy [25,26]. For example, the wavelet neural network fully inherits the excellent time-frequency localization characteristics of the wavelet transform. It has the self-learning characteristics of the neural network, so it has nonlinear solid approximation capability [27,28]. Wang et al. [29] proposed a model-free SOH estimation method based on discrete wavelet transform. The method’s feasibility was proved by analyzing the output results of different phenomena through a dynamic stress test on the battery. However, the traditional wavelet neural network is trained primarily by the subtraction method, which has the defect of a local optimum. The fusion method can overcome the shortcomings of a single algorithm and give full play to the advantages of multi-algorithm fusion. Jia et al. [30] proposed a multi-scale SOH prediction method, which combined the wavelet neural network and untraced particle filter model and decomposed into low-frequency attenuation trend and high-frequency fluctuation components through discrete wavelet transform. Low-frequency degradation trend data were used to predict the SOH of lithium-ion batteries. Experimental results show that the proposed method still has high accuracy and strong robustness in the early stage of battery life problems. Although the fused neural network method is ideal in battery SOH estimation, the network model needs to be trained under a large amount of data, so extracting high-quality features to make the model have good performance is also a fundamental problem.

The deep learning method does not require a complex feature extraction process and has a strong learning ability and strong generalization of the algorithm [31,32,33]. As an algorithm structure, it is characterized by multiple parameters and layers, including convolutional neural networks [34], recursive neural networks [35], and other network structures. With the development of 5G technology and computer technology, powerful data computing power can be used to make up for the lack of algorithm complexity, so more deep learning algorithms are used for health state estimation and remaining service life prediction of lithium-ion batteries.

The deep convolutional neural network (DCNN) has stacked the single-layer convolutional neural network many times. By layering layers of hidden layers, the previous layer’s output is used as the input of the last layer. This simple model can be used to complete the target learning task and significantly reduce the accuracy error of the calculation results [36]. Su, Laisuo et al. [37] compared the convolutional neural network with the traditional neural network model, used multi-layer convolution to capture the hidden features of lithium-ion batteries from the voltage distribution map, and proved that these hidden features had higher covariance with their cycle life, and the prediction accuracy was also extremely improved. Fan [38] adopted an innovative modeling method; A hybrid algorithm based on a gate-recursive element convolutional neural network was proposed to analyze and study the charging voltage curve of lithium batteries. It provides a novel method for SOH estimation and life prediction. Tracing information from early battery charge measurements, for instance, voltage, current, and temperature, is used to estimate SOH online, Through the verification of two datasets, the effect is also considerable. However, its disadvantages are evident, mainly requiring many training samples and the algorithm’s complexity, which requires the system to have a high computing capacity. 

However, the aging of the battery is a time series transform process, and historical data are also a considerable feature of information. A recursive neural network (RNN) adopts a feedback mode to return the output parameters to the input and transmit the information back to the network, completing a cycle. Therefore, the network model can remember historical data and apply it to prediction [39]. Nonetheless, when the effective information interval is long, the backpropagation of the RNN will produce the phenomenon of gradient disappearance or explosion. To improve the performance of the model, researchers modify the original neurons and create a more complex classical structure, The long short-term memory recurrent neural network, whose characteristics can be well applied to the estimation and prediction of battery SOH.

**Figure 1:**
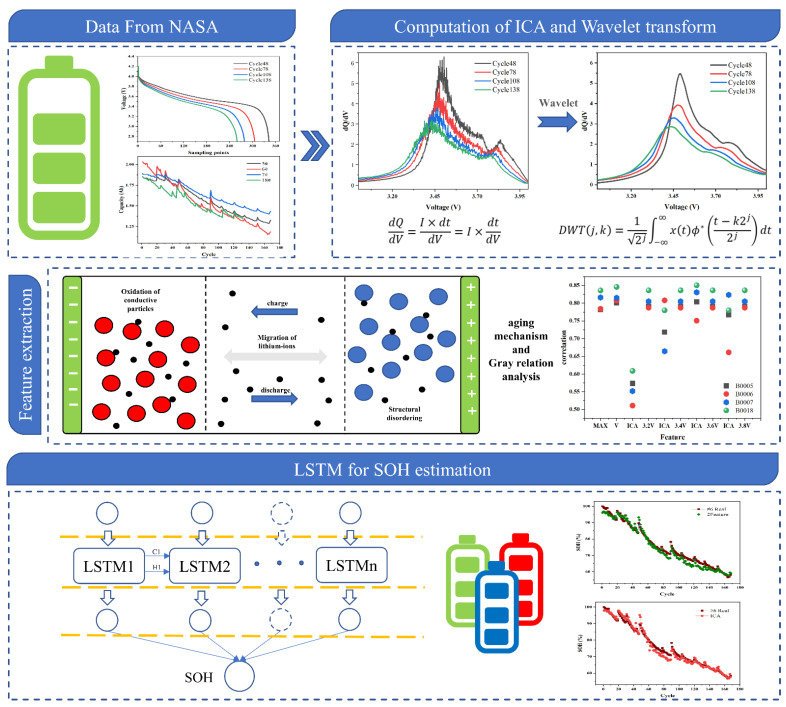
Flow diagram of SOH estimation.

Most studies show that one of the current obstacles to lithium battery management systems is a degradation of battery health status, which is mainly reflected in capacity loss [40]. However, the battery’s aging does not directly manifest itself in the attenuation of capacity during the early cycle but will affect the voltage curve of the early cycle discharge [41]. Therefore, in order to better improve the SOH prognostics accuracy of lithium-ion batteries, a deep learning method combined with mechanism analysis is proposed to optimize the input of deep learning by mining the early discharge voltage data. The specific prediction flow chart of SOH is shown in Figure 1.

The rest of this article is organized as follows: Section 2 introduces the experimental battery dataset and incremental capacity analysis methods. In Section 3, the structure of the LSTM model was introduced, and the battery health state estimation model was established. Section 4 presents the result of the battery SOH estimation. The conclusion is put in Section 5.

## 2. Data Preprocessing

This section mainly analyzes the battery aging data from NASA and preprocesses the aging data. At first, the data were cleaned by the kernel smoothing method, and the original incremental capacity value was obtained by calculating the relation between dQ/dV and V. It was found that the influence of noise was inevitable by observing the data. The advanced discrete wavelet transform was used to filter the interference brought by noise and prepared for the subsequent analysis of the aging mechanism.

### 2.1. Data Acquisition

**Figure 2:**
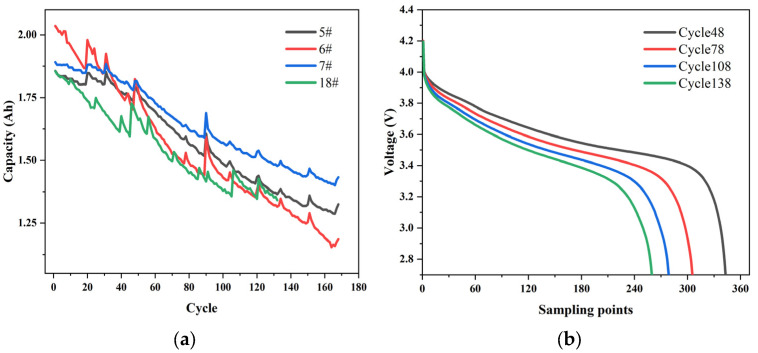
Battery aging process and discharge voltage variation: (**a**) capacity attenuation curve; (**b**) voltage variation of the aging cycle schemes follow another format.

This article selected datasets from the NASA battery Prediction test platform. Datasets B0005, B0006, B0007, and B0018 were selected to obtain the aging trend of battery life under different conditions. These data are run through four different operation data at room temperature (24 degrees Celsius). Responsible for performing charging in 1.5 A constant current (CC) mode until battery voltage reaches 4.2 V, then continuing in constant voltage (CV) mode until charging current drops to 20 mA. The four batteries are discharged at a constant current 2 A level while waiting for the voltage of 5#, 6#, 7#, and 18# batteries to drop to 2.7, 2.5, 2.2, and 2.5 V, respectively. When the capacity of the battery is lower than 30% of the rated capacity after several cycles, that is, it reaches the end of life, and the experiment stops. These datasets can predict battery SOH. The specific charging and discharging conditions of the four batteries are shown in Table 1. The aging of the battery tends with the number of cycles shown in Figure 2a, Figure 2b indicates the voltage variation of an aging cycle for battery5#.

As shown in the figure, battery aging does not show capacity attenuation during the early cycle but will affect the early cycle discharge voltage curve. The voltage curve and its derivative are a rich data source, which is very effective in aging diagnosis. The characteristics obtained from the early discharge voltage curve have good predictive performance, even before the decline in battery capacity begins. Therefore, we use the voltage of the discharge cycle to calculate the incremental capacity (IC) curves and extract some features from the IC curve to build a high-precision battery prediction model [38].

### 2.2. Increment Capacity Curve Analysis

Incremental capacity analysis (ICA) is an important approach to studying the degradation mechanism of material properties of lithium-ion power batteries. The increment capacity curve obtained from the voltage and current data during the charging and discharging process can well reflect the changes in the internal chemical characteristics of lithium-ion power batteries.

In this paper, using the relationship between capacity and voltage in the discharge process to conduct increment capacity analysis, the calculation can be obtained as follows:

(1) $$\frac{dQ}{dV}=\frac{I\times dt}{dV}=I\times \frac{dt}{dV}$$

where *Q* is the ampere-hour of discharge, and *V* is the voltage in the discharge stage. Additionally, the constant discharge current is 2 A. The ICA curve is computed by Equation (1). There are many noises in the signal, which brings specific difficulties for subsequent feature extraction work, so we need to use advanced filtering methods to obtain a smoother curve.

The variation trend of the effective signal is generally stable, and the frequency is mainly gathered in the low-frequency band. The noise or useless signal change generally has great uncertainty or fluctuation, and the frequency is primarily in the high-frequency band. So, we will use the method of wavelet noise filtering to process the ICA curve. 

The DWT can be obtained by discrete scaling and shift parameters through the Mallat algorithm, which primarily operates a pair of low-pass and high-pass wavelet filters. The signal is reconstructed by the selected decomposition scale and the corresponding wavelet basis function. Finally, the wavelet transform method is used to decompose and reconstruct the signal.

We use discrete wavelet transform to capture nonstationary feature information. When the *ϕ*(t) ∈ *L*^2^(*R*) with zero bases, the DWT can be defined as:

(2) $$DWT\left(j,k\right)=\frac{1}{\sqrt{2^{j}}}\int_{-\infty }^{\infty }x\left(t\right)\phi^{*}\left(\frac{t-k2^{j}}{2^{j}}\right)dt$$

where *ϕ*(*t*) is called the fundamental wavelet and the asterisk indicates the complex conjugate. In Equation (2), there are two parameters of dilation *j* and translation *k*. The parameter *j* impacts the oscillatory frequency and the length of the wavelet. The moved position can be ensured by the parameter *k*.

**Figure 3:**
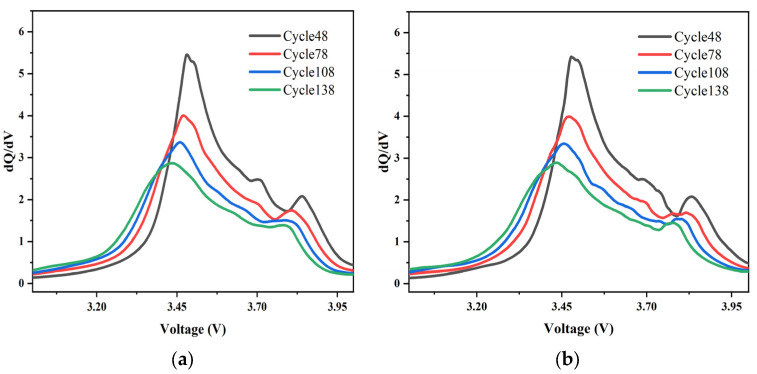

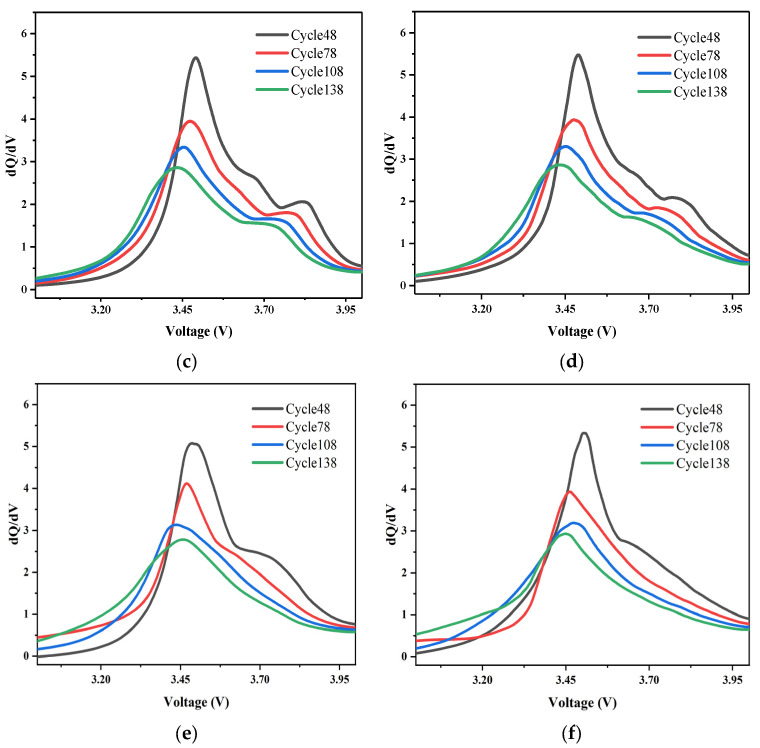
ICA curves after different wavelet filtering. (**a**) Layers:5, wave:sym6. (**b**) Layers:5, wave:db4. (**c**) Layers:6, wave:sym6. (**d**) Layers:6, wave:db4. (**e**) Layers:7, wave:sym6. (**f**) Layers:7, wave:db4.

The effect of different decomposition levels and different fundamental selections is shown in Figure 3, too many decomposition layers will distort the voltage signal and thus reduce the accuracy. Too few decomposition levels do not do a good job of culling the effects of noise. Horizontal comparison can be seen as the best effect is achieved when the decomposition layers are 6.

There is no theoretical standard for the choice of wavelet basis functions. No wavelet basis function can be optimally denoised for a variety of signals. The Daubechies wavelet family is one of the typical discrete wavelet families and is often used for denoising due to its orthogonality and tight support. As can be seen from the local decomposition diagram, the same 6-layer decomposition, the consistency of fundamental wave selection sym6 is poor, and the db4 total effect is much better by using the Daubechies wavelet family. So by longitudinal comparison, the db4 wavelet basis function is chosen.

## 3. Methodologies

### 3.1. Grey Relation Analysis

It can be seen in Figure 3 that there is a noticeable peak on the discharge incremental capacity curve, and the district under each peak represents the capacity of the related reaction. The aging mechanism of the battery can be determined by analyzing the change of the increase in each peak with the number of cycles.

As the number of cycles continues to increase, the peak value of the IC curve decreased significantly, indicating that the loss of active material, especially anode material, caused peak degradation. In addition, the change in the peak position means that the battery resistance has also changed. All the peak values of the IC curve move towards low voltage, indicating that the battery resistance is gradually decreasing.

Then, the gray relation analysis method provides a quantitative measurement method for the situation in the development and change of the system and is appropriate for dynamic history analysis. it can judge the correlation between curves by comparing the similarity degree of curve changing trend. Grey relation analysis is a new method to analyze sequence correlation, which can make a good evaluation of the correlation between sequences even in the case of small samples or poor sample information. 

The calculation of gray relation analysis are as follows:

(1) Collect the original sequence and select the comparison sequence Xi and reference sequence Y:

(3) $$X_{i}=\{x_{i}\left(k\right)|k=1,2,\cdots ,n\}$$

(4) Y={y(k)|k=1,2,⋯,n}

where *n* represents the size of the comparison sequence and the reference sequence. “i = 1,2,…,*m*”, *m* is the number of comparison sequences.

(2) Calculate the correlation coefficient *ξ_i_ (k)* between *x_i_ (k)* and *y_i_ (k)*:

(5) $$\xi_{i}\left(k\right)=\frac{\begin{array}{c} \min\\ i \end{array}\begin{array}{c} \min\\ k \end{array}|y\left(k\right)-x_{i}\left(k\right)|+\rho \begin{array}{c} \max\\ i \end{array}\begin{array}{c} \max\\ k \end{array}|y\left(k\right)-x_{i}\left(k\right)|}{|y\left(k\right)-x_{i}\left(k\right)|+\rho \begin{array}{c} \max\\ i \end{array}\begin{array}{c} \max\\ k \end{array}|y\left(k\right)-x_{i}\left(k\right)|}$$

(6) $$a=\begin{array}{c} \min\\ i \end{array}\begin{array}{c} \min\\ k \end{array}|y\left(k\right)-x_{i}\left(k\right)|,b=\begin{array}{c} \max\\ i \end{array}\begin{array}{c} \max\\ k \end{array}|y\left(k\right)-x_{i}\left(k\right)|$$

where *ρ* ∈ [0, 1] is the resolution coefficient, the value is usually set to 0.5, a and b are the minimum and maximum polar differences of the reference sequence and the comparison sequence, respectively.

(3) The related degree *r_i_* between reference sequence *Y* and comparison sequence *x_i_* is calculated:

(7) $$r_{i}=\frac{1}{n}\sum_{k=1}^{n}\xi_{i}\left(k\right),k=1,2,\cdots ,n$$

where *r_i_* ∈ [0, 1], the closer the correlation degree *r_i_* is to 1, the greater the correlation between *X_i_* and *Y.*

The feature of peak ICA and its corresponding voltage, ICA value corresponding to 3.2 V, ICA value corresponding to 3.4 V, ICA value corresponding to 3.6 V, and ICA value corresponding to 3.8 V as the input of gray correlation comparison sequence, and capacity as the input of gray correlation reference sequence. The results obtained are shown in Table 2.

F1, F2 is the peak ICA value corresponding to the voltage, and F3–F6 is the ICA value corresponding to the above voltage. Since the voltage does not change, the characteristics corresponding to the fixed voltage are no longer listed.

As dQ/dV-V is a set of correspondence, it can be seen from Table 2 that peak ICA and its corresponding voltage are both high, while other extracted features have high voltage correlation and IC value correlation. To verify the feasibility, the four features with the highest correlation are taken as the first group, and peak ICA is taken as the second group. Peak ICA and its voltage are the third set of characteristic inputs for subsequent models.

### 3.2. Long Short-Term Memory Modeling

RNN is a network with an inherent loop that processes sequences by iterating through all sequence elements and preserving states containing time-step history feedback.

**Figure 4:**
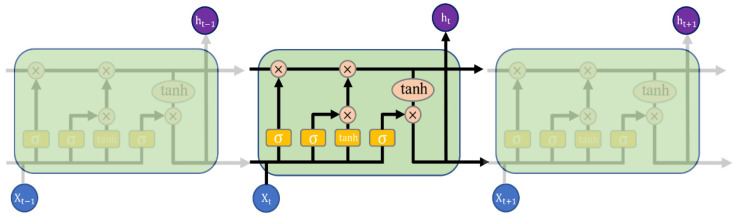
LSTM structure.

LSTM is a special RNN that effectively mitigates gradient disappearance and gradient explosion with its’ gating mechanism. The internal structure of the general LSTM neural network is shown in Figure 4. The various elements of the LSTM are displayed below.

(8) $$f_{t}=\sigma \left(W_{xf}x_{t}+W_{hf}h_{t-1}+b_{f}\right)$$

(9) $$i_{t}=\sigma \left(W_{xi}x_{t}+W_{hi}h_{t-1}+b_{i}\right)$$

(10) $$o_{t}=\sigma \left(W_{xo}x_{t}+W_{ho}h_{t-1}+b_{o}\right)$$

(11) $$c_{t}=f_{t}\cdot c_{t-1}+i_{t}\cdot tanh\left(W_{xc}x_{t}+W_{hc}h_{t-1}+b_{c}\right)$$

(12) $$h_{t}=o_{t}\cdot tanh\left(c_{t}\right)$$

where *x_t_* is the input data comprised of increment capacity curve peak and corresponding voltage, subscript t indicates the time step, among them, *i*, *f,* and *o* are three gates, representing input gate, forgetting gate, and output gate, respectively. Long short-term memory can voluntarily add or forget information through the input gate and forget gate, *W*, *b* are the weights and biases. The activation function is represented as *σ*, The sigmoid function is often used to adjust its output value and limit it to values between 0 and 1. When generating candidate memory, the activation function selects tanh to accelerate the convergence of the model. The battery SOH estimation model based on LSTM in this article is based on TensorFlow in Python, which is a commonly used deep learning framework.

## 4. Results and Discussion

### 4.1. Model Training Structure

Through the constant adjustment of the model structure, it was found that the number of LSTM layers should not be too high, as the growth in the number of layers will lead to an exponential increase in time and memory overhead, followed by gradient disappearance between layers. When the number of LSTM layers exceeds three or more layers, the gradient disappearing between layers seems very distinct. Because of the time series model, the update iteration of the LSTM layer near the input layer becomes slow. The efficiency of model convergence will also decrease sharply, and it is light to enter the dilemma of local minimum. Therefore, the use of two layers of LSTM in this article maintains a relatively good effect.

Table 3 lists the structure and some of the hyperparameters of the LSTM model. Due to the aging through the mechanism analysis of characteristics and SOH highly correlated, two layers of hidden units, respectively, 75 the establishment of LSTM layer to reach a satisfactory estimation precision, training process, the loss was calculated by the mean absolute error function is more advantageous to regression problems, and use the “Adam” optimizer training network, in order to avoid excessive fitting. Using the Dropout layer, 50% of the training samples are randomly dropped, and root mean square error (RMSE) and mean absolute error (MAE) are used to define the loss function.

### 4.2. Estimation Results of the Model

**Figure 5:**
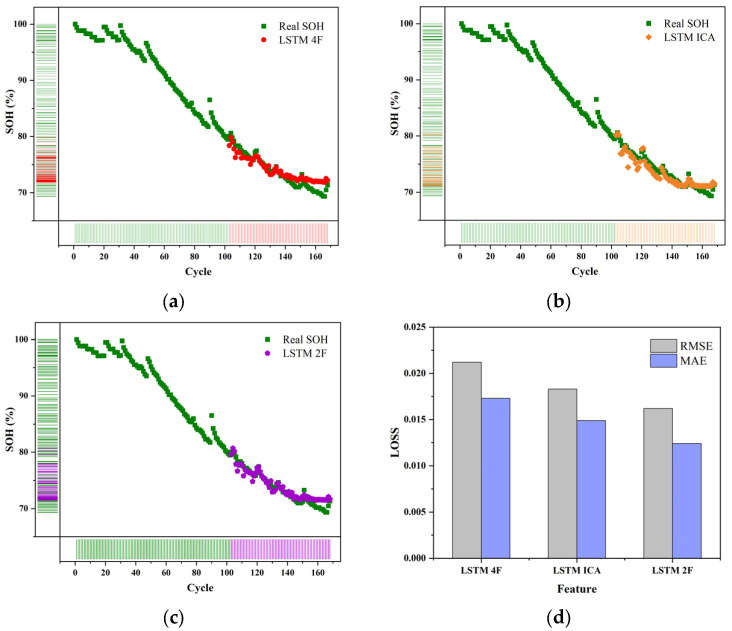

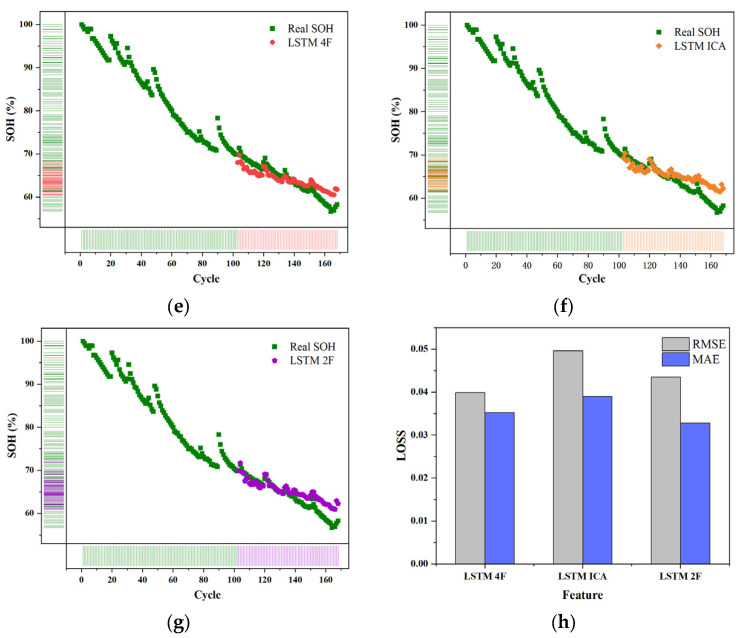
LSTM for 5# and 6# batteries (**a**–**c**). The predicted results of different features for 5#. (**d**) The loss of battery 5# (**e**–**g**). The predicted results of different features for 6# (**h**). The loss of battery 6#.

First, the prediction model of the single-cell model was established. By observing the capacity attenuation curve and others’ analysis of the dataset, we could know that the EOL of the four batteries was all after 100 cycles. Therefore, we took the first 100 cycles as the training set and the remaining cycles as the test set to train single batteries. The predicted curves and loss rates of different characteristics are shown in Figure 5.

Through observation, it was found that the initial capacity of the four batteries is less than the rated capacity, indicating that the battery has aged before the test, so in this article, the capacity of the initial cycle is used as a reference to calculate, through the training of the 5# battery, it is found that the overall loss is less than 3%, so the effect is satisfactory.

There are three groups of different features in the input, the ICA-V prediction effect of this group is the best, and the loss rate is the lowest, although the loss of the three groups was lower than 2.5%. Additionally, it can also obtain good results under the single-peak feature, indicating that the results of gray relation analysis have been proved.

Then, we turn our attention to the 6# battery, it was found that the loss was higher than the other three batteries, and it can be seen through the conversion calibration that the SOH of the battery dropped below 80% during the first 60 cycles alone, indicating that the aging of the 6# battery has been very serious.

**Figure 6:**
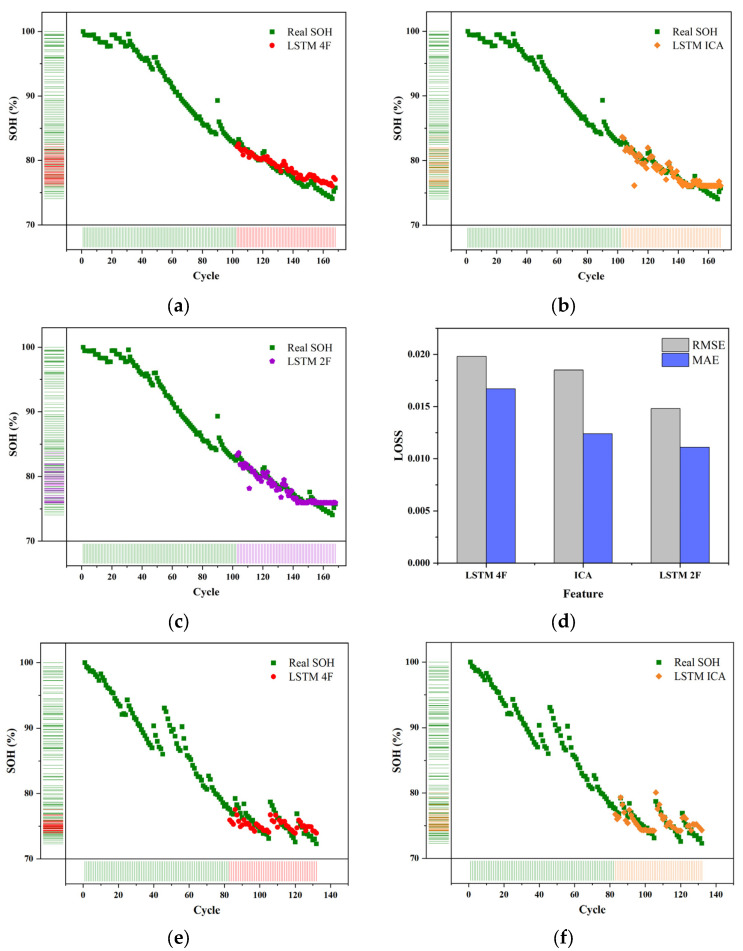

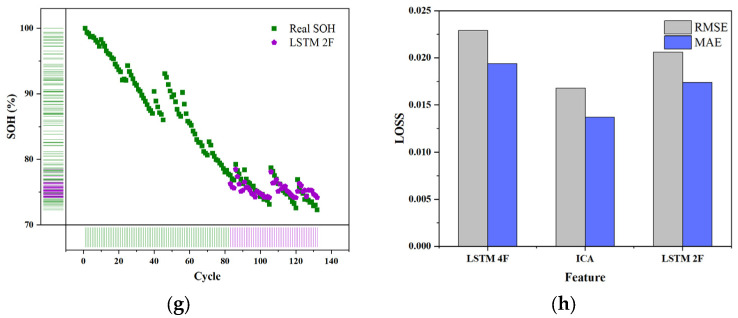
LSTM for 7# and 18# batteries (**a**–**c**). The predicted results of different features for 7# (**d**). The loss of battery 7# (**e**–**g**). The predicted results of different features for 18# (**h**). The loss of battery 18#.

After adjusting the model structure to a certain extent, the object is converted into the remaining 6#, 7#, and 18# batteries, the loss rate of the test set is all less than 5%, especially on the 5#, 7#,18# battery, the loss rate is less than 2.5%, the predicted results are shown in Figure 6, and the specific verification data are shown in Table 4 and Table 5, which further verify the accuracy of the model and determine that the model has a particular generalization ability.

### 4.3. Estimation Results of the Model

Since the previous prediction of the 6# battery was not as good as the other three batteries, the article then used transfer learning to improve the prediction accuracy of the 6 batteries.

**Figure 7:**
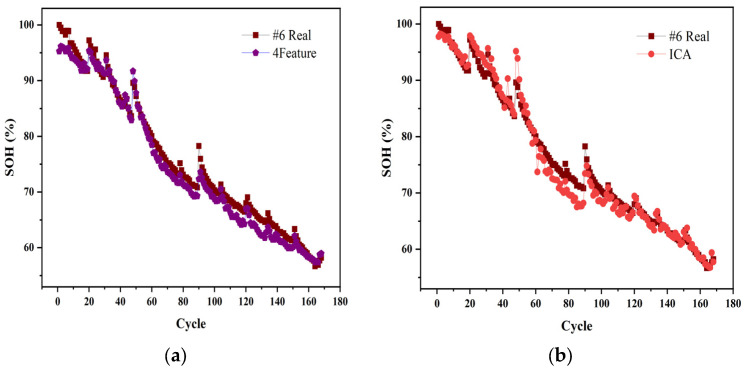

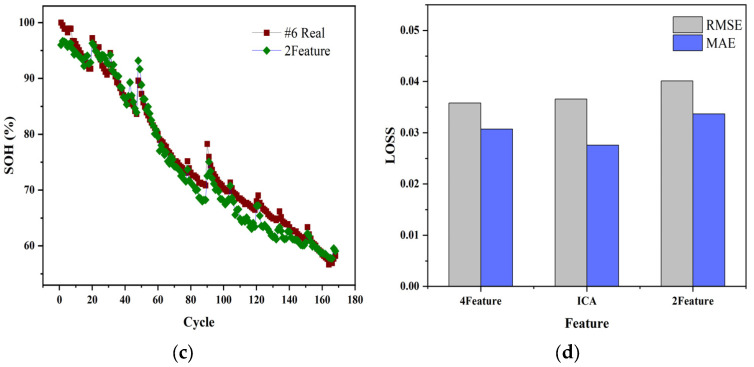
Transform learning for 6# battery (**a**–**c**). The predicted results of different features for 6# (**d**). The loss of battery 6#.

The neural network framework with the same structure as the previous article is built, the first two layers of the LSTM recurrent network are set to the frozen state, the last two layers are assigned to the trainable state, all the aging data of the 5# battery are used as the training set, and the test set is set to the whole sequence of the 6# battery, the prediction effect of the model is shown in Figure 7. It can be seen through the training of this mode, the modified model can predict other batteries similar to the loading mode. Additionally, the average loss rate of different feature inputs has dropped to approximately 3.5%, which verifies the effectiveness of the migration model.

**Figure 8:**
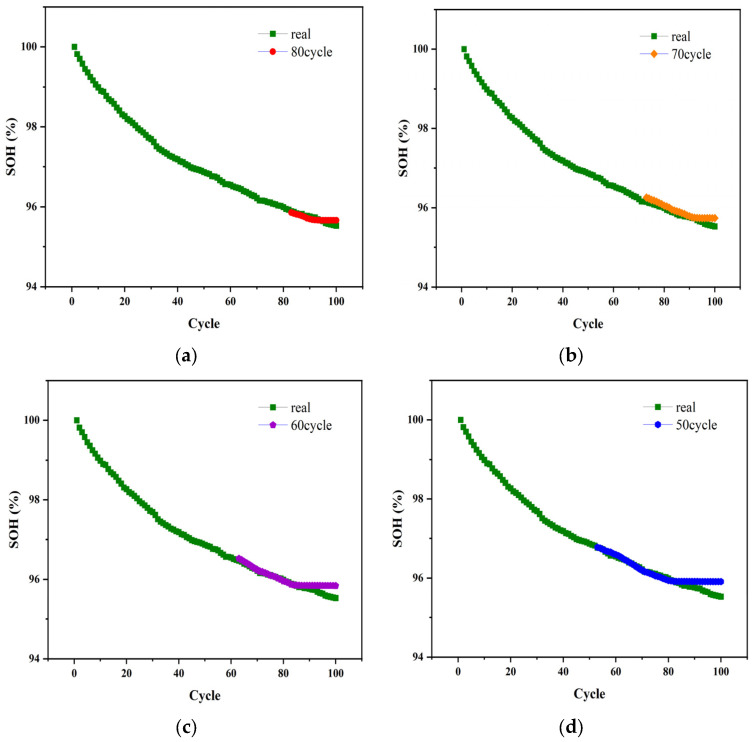
Transfer learning on the Mendeley dataset. (**a**) 80% of the training data. (**b**) 70% of the training data. (**c**) 60% of the training data. (**d**) 50% of the training data.

Otherwise, as shown in Figure 8, without changing the model structure, the model was migrated to the Mendeley dataset, and the model accuracy was tested when the first 50%, 60%, 70%, and 80% of the training data were taken from the overall battery data. The results are shown in Table 6, indicating that the modified model can accurately assess the health status of lithium-ion batteries in the Mendeley dataset.

## 5. Conclusions

Accurately predicting and estimating the SOH of the battery system is essential to achieving reliable, efficient, and affordable batteries. The challenge in lithium-ion battery SOH prediction is primarily how to accurately recognize the long-term correlation of hundreds of cycles of batteries based on limited aging data. This paper introduces a SOH prognostic method combining aging mechanism analysis and deep learning. It can still maintain good accuracy in the case of only small batch data. The specific contributions are as follows. 

In order to ensure the validity of the data and the accuracy of the subsequent calculation results, kernel smoothing methods are used to remove the outliers when the NASA dataset is preprocessed. Additionally, through the calculation and analysis of the voltage curve, a capacity increment curve that can characterize the aging characteristics of the battery is obtained.

Due to the influence of noise, the observation of signal characteristics is not obvious, and a discrete wavelet transform of decomposition and reconstruction is used to capture the signal characteristics and filter the effects of noise. By analyzing the aging mechanism of the increment capacity curve, a distinct group based on the peak of the curve and the corresponding voltage was extracted. Different from the extraction of other model features, incremental capacity curves with large information features are analyzed and extracted by GRA, combined with a battery aging mechanism to optimize the input of deep learning models. The verification results show that the SOH estimation model has good generalization ability and high prediction accuracy, and the MAE and RMSE of the predicted results are 1.24% and 1.62%, respectively. The error is less than 5% in the subsequent training process of battery 6#. In order to verify the model’s mobility, the effect is acceptable by freezing the LSTM layers, slight adjustments to the remaining structure are used for other battery training, and the error is less than 4%.

Owing to the uncertainty of the set weights of deep learning random numbers, the network recommendations compared during the technical evaluation should have a similar number of learnable parameters and use the exact data for training and testing. Otherwise, it is difficult to make general conclusions about the estimated quality. In addition, there are multitudinous environmental factors and the influence of the model itself, such as different loading modes, different working conditions, and ambient temperature. The LSTM network cannot solve difficult parallel computing problems, so the practicability of the model needs to be further considered.

## Figures and Tables

**Table 1:** Experiment condition of the battery.

Battery Number	Discharge Current	Voltage Upper	Voltage Lower
B0005	2 A	4.2 V	2.7 V
B0006	2 A	4.2 V	2.5 V
B0007	2 A	4.2 V	2.2 V
B0018	2 A	4.2 V	2.2 V

**Table 2:** Grey relation analysis.

Battery Number	Grey Relation Coefficient					
	F1	F2	F3	F4	F5	F6
5#	0.7815	0.8009	0.5735	0.7184	0.8036	0.7674
6#	0.7842	0.8074	0.5109	0.8081	0.7506	0.6611
7#	0.8161	0.8151	0.5519	0.6642	0.8308	0.8235
18#	0.8361	0.8459	0.6090	0.7801	0.8506	0.7799

**Table 3:** Hyperparameters setting of LSTM model.

Parameters	Value
Number of units in the LSTM 1	75
Number of units in the LSTM 2	80
Dense	25
Dropout	0.5
Dense	1

**Table 4:** The loss rate of 5# and 6#.

	B0005			B0006		
	LSTM 4F	LSTM ICA	LSTM 2F	LSTM 4F	LSTM ICA	LSTM 2F
RMSE	0.0212	0.0183	0.0162	0.0399	0.0496	0.0435
MAE	0.0173	0.0149	0.0124	0.0352	0.0390	0.0328

**Table 5:** The loss rate of 7# and 18#.

	B0007			B00018		
	LSTM 4F	LSTM ICA	LSTM 2F	LSTM 4F	LSTM ICA	LSTM 2F
RMSE	0.0198	0.0185	0.0148	0.0229	0.0168	0.0206
MAE	0.0167	0.0124	0.0111	0.0194	0.0137	0.0174

**Table 6:** Comparison of prediction results of different training scale models.

Train Scale (%)	RMSE	MAE
80	0.0174	0.0146
70	0.0269	0.0236
60	0.0319	0.0208
50	0.0381	0.0248

## Data Availability

https://ti.arc.nasa.gov/tech/dash/groups/pcoe/prognostic-data-repository/#battery.
